# Host Responses to Melioidosis and Tuberculosis Are Both Dominated by Interferon-Mediated Signaling

**DOI:** 10.1371/journal.pone.0054961

**Published:** 2013-01-29

**Authors:** Gavin C. K. W. Koh, M. Fernanda Schreiber, Ruben Bautista, Rapeephan R. Maude, Susanna Dunachie, Direk Limmathurotsakul, Nicholas P. J. Day, Gordon Dougan, Sharon J. Peacock

**Affiliations:** 1 Wellcome Trust Sanger Institute, Hinxton, Cambridge, United Kingdom; 2 Department of Medicine, University of Cambridge, Cambridge, United Kingdom; 3 Mahidol-Oxford Tropical Medicine Research Unit, Mahidol University, Bangkok, Thailand; 4 Department of Infection and Tropical Diseases, Birmingham Heartlands Hospital, Birmingham, United Kingdom; 5 Department of Microbiology, Mahidol University, Bangkok, Thailand; Institut de Pharmacologie et de Biologie Structurale, France

## Abstract

Melioidosis (*Burkholderia pseudomallei* infection) is a common cause of community-acquired sepsis in Northeast Thailand and northern Australia. *B. pseudomallei* is a soil saprophyte endemic to Southeast Asia and northern Australia. The clinical presentation of melioidosis may mimic tuberculosis (both cause chronic suppurative lesions unresponsive to conventional antibiotics and both commonly affect the lungs). The two diseases have overlapping risk profiles (e.g., diabetes, corticosteroid use), and both *B. pseudomallei* and *Mycobacterium tuberculosis* are intracellular pathogens. There are however important differences: the majority of melioidosis cases are acute, not chronic, and present with severe sepsis and a mortality rate that approaches 50% despite appropriate antimicrobial therapy. By contrast, tuberculosis is characteristically a chronic illness with mortality <2% with appropriate antimicrobial chemotherapy. We examined the gene expression profiles of total peripheral leukocytes in two cohorts of patients, one with acute melioidosis (30 patients and 30 controls) and another with tuberculosis (20 patients and 24 controls). Interferon-mediated responses dominate the host response to both infections, and both type 1 and type 2 interferon responses are important. An 86-gene signature previously thought to be specific for tuberculosis is also found in melioidosis. We conclude that the host responses to melioidosis and to tuberculosis are similar: both are dominated by interferon-signalling pathways and this similarity means gene expression signatures from whole blood do not distinguish between these two diseases.

## Introduction

Melioidosis is a serious infectious disease caused by the environmental Gram-negative bacillus *Burkholderia pseudomallei*. The organism is distributed in soil across much of southeast Asia and northern Australia [1], and infection results from bacterial inoculation, ingestion or inhalation of the organism. The majority of cases are reported during the rainy season in northeast Thailand and northern Australia, and the most common presenting features are community-acquired pneumonia and/or bacteraemia [1]. The spectrum of clinical manifestations is very diverse, and melioidosis may present as acute, chronic and latent disease. Patients with chronic melioidosis may present with signs, symptoms and chest radiograph changes that are indistinguishable from tuberculosis [1].

Comparisons have also been drawn between melioidosis and tuberculosis based on apparent parallels in epidemiology, pathophysiology and therapy. Melioidosis and tuberculosis have risk factors in common, in that corticosteroid use and diabetes predispose to both diseases [1]. At clinical presentation, chronic melioidosis, like tuberculosis, causes chronic suppurative lesions that do not respond to commonly used first line antimicrobials (viz., aminopenicillins, first and second generation cephalosporins, macrolides or aminoglycosides). Histological examination of tissue taken from patients with melioidosis may reveal granulomas with central necrosis, which mimic those of tuberculosis [2]. At a cellular level, both *B. pseudomallei* and *Mycobacterium tuberculosis* are intracellular pathogens and this ability to parasitise cells appears crucial to their virulence [3], [4]. Melioidosis, like tuberculosis, is also able to cause latent infection, the longest documented interval between exposure and clinical melioidosis being 62 years [1].

While chronic melioidosis is clinically similar to active tuberculosis, and latent of forms of both melioidosis and tuberculosis undoubtedly occur, acute melioidosis has no clinical counterpart in tuberculosis. Only ∼10% of melioidosis cases are chronic (symptoms >2 months) [1], and the majority of melioidosis cases present acutely, with sepsis frequently complicated by hypotension and organ dysfunction, which rarely occurs in tuberculosis. Acute melioidosis is therefore a clinical entity quite distinct from tuberculosis. In northeast Thailand, mortality is 40% despite appropriate treatment [5], whereas tuberculosis mortality is <2% with appropriate treatment. HIV infection is also an important risk factor for tuberculosis, but there is no established association between HIV and melioidosis [6]. The taxonomic relationship between *B. pseudomallei* and *M. tuberculosis* is distant (they are in different phyla: Proteobacteria and Actinobacteria, respectively). Their cell surfaces also present different pathogen-associated molecular patterns (PAMP) to the host immune system, and it seems reasonable to expect the host to respond differently to challenge by different PAMPs.

In this study, we sought differences in host response between acute melioidosis and tuberculosis using whole genome arrays to compare gene expression in circulating peripheral blood leukocytes collected from two cohorts of patients, one with melioidosis and one with tuberculosis. We also sought to define whether whole blood gene expression profiling distinguishes between melioidosis and tuberculosis.

## Materials and Methods

The melioidosis data were taken from a previously published cohort of 30 patients and 30 healthy controls, frequency-matched for diabetes and glibenclamide use (an oral hypoglycaemic drug used to treat diabetes mellitus) [5]. Each group contained 10 non-diabetics and 20 diabetics. Diabetics were divided into 10 taking glibenclamide ( = glyburide) and 10 not taking any sulphonylurea (but who may have been on insulin, metformin or diet-control alone). We adjusted for diabetes and glibenclamide because two-thirds of all melioidosis patients have diabetes, diabetes is itself a pro-inflammatory condition, and because glibenclamide is anti-inflammatory [5]. The tuberculosis cohort has been published previously and consists of 20 patients with pulmonary tuberculosis and 24 healthy controls [7]. That study did not control for the effect of confounders such as diabetes. Inclusion and exclusion criteria for both studies have been published previously [5], [7]. Eligible cases for both studies were persons aged between 18 and 75 years. In the melioidosis cohort, diabetes was defined as an abnormal Hb A_1c_ at enrolment or a previous diagnosis of diabetes. The tuberculosis cohort excluded patients with diabetes. Both studies excluded patients who were pregnant or immunosuppressed.

### Melioidosis Microarrays

The methods used in the melioidosis cohort have been reported previously [5] and the data is deposited at ArrayExpress, EMBL-EBI (accession number E-TABM-852-n). In brief, a 3 ml blood sample was collected from each study subject in a PaxGene™ Blood RNA tube (PreAnalytiX, GmbH) and stored at –70°C. RNA was extracted using the PaxGene™ Blood RNA Purification Kit (PreAnalytix) according to the manufacturer’s instructions. The RNA was amplified using the Illumina® TotalPrep RNA Amplification Kit (Applied Biosystems) and assayed using the Illumina® HumanWG-6 v3.0 Expression BeadChip (Illumina®), which probes 48,803 transcripts from across the human genome. Quantitative PCR verification of these microarrays has been reported previously [5].

### Tuberculosis Microarrays

The methods used in the tuberculosis cohort have been published elsewhere previously [7]. In brief, a 3 ml blood sample was collected into Tempus tubes (Applied Biosystems, California) and stored at –20 to –80°C. RNA was extracted using the PerfectPure RNA Blood Kit (5 PRIME) according to the manufacturer’s instructions. The RNA was then amplified using the Illumina CustomPrep RNA amplification kit (Applied Biosystems) and assayed using the Illumina Human HT-12 v3 BeadChip array (Illumina), which uses the same probe set as the HumanWG-6 v3.0. Raw data was downloaded from a publicly available repository (NCBI GEO accession number GSE19491) and consists of tuberculosis patients with controls recruited in London. The study also included data from a cohort of South African tuberculosis patients, but that cohort was excluded from this analysis because it does not contain uninfected controls, which made it impossible to normalize across the cohorts. The original study was analysed using GeneSpring, but we reanalysed the raw data using Bioconductor for the sake of comparability.

### Ethics

Approval for the melioidosis study was obtained from the Oxford Tropical Research Ethics Committee (OXTREC 018-07) and the Ethics Committee of the Faculty of Tropical Medicine, Mahidol University (MUTM 2008-001-01) [5]. Approval for the tuberculosis study was obtained from the Research Ethics Committee at St Mary’s Hospital, London, UK (REC 06/Q0403/128) [7]. Written informed consent was obtained from all subjects.

### Statistical Methods

Differential expression analyses were performed using Bioconductor [8] version 2.12.1 running on R 2.13.0 [9]. Pre-processing was performed using the beadarray 2.2.0 package [10], [11], and background correction was performed using normexp [12], [13]. Fluorescence intensities were quantile-normalized between arrays within each cohort and non-expressed probes were removed (detection *p*-value >0.05). Differential expression was performed using limma 3.8.1 [14]. For the melioidosis cohort, the linear model fit was log *e* = *β*_0_ + *β*_1_ *x*_1_ + *β*_2_ *x*_2_ + *β*_3_ *x*_3_ + *β*_12_ *x*_1_ *x*_2_, where *e* is expression, *x*_1_ is melioidosis, *x*_2_ is diabetes and *x*_3_ is glibenclamide treatment. The expression values for melioidosis, *β*_1_, are therefore adjusted for diabetes and glibenclamide treatment. For the tuberculosis data, the model was log *e* = *β*_0_ + *β*_1_ *x*_1_, where *x*_1_ is tuberculosis. The *p*-value cut-off of 0.01 was set following visual inspection of the histogram of unadjusted *p*-values for *β*_1_, as calculated from the moderated *t*-statistic (B-statistic) using Bayesian methods [14]. Illumina probe IDs were mapped to HUGO gene symbols [15] by illuminaHumanv3.db [16]. Networks were clustered by pathway by the Reactome [17] functional interaction network [18] plug-in for Cytoscape 2.8.1 [19], restricting the analysis to modules larger than 10 proteins. The *p*-values reported are for the hypergeometric test. The top 1000 probes were used to construct networks for presentation in figures. We searched specifically for interferon-regulated gene signatures on Interferome also [20]. Heat maps were drawn with gplots 2.8.0 [21] using colour blind-safe colour ramps generated by RColorBrewer 1.0–2 [22], [23]. We divided controls and patients by unsupervised *k*-means [24] and verified stability of the clusters under 5 random starts.

## Results

The melioidosis cohort consisted of 30 patients and 30 controls. Baseline characteristics are in Table 1. In the melioidosis cohort, 6,755 probes were differentially expressed (that is, either up or downregulated) representing 4632 unique genes. Annotation was available for 1,658 of these genes, of which 651 were upregulated and 1,007 were downregulated. The tuberculosis cohort consisted of 20 patients and 24 controls. In the tuberculosis cohort, 6911 probes differentially expressed (5045 unique genes). Annotation was available for 1985 of these genes, of which 847 were upregulated and 1138 were downregulated. In both the melioidosis and the tuberculosis cohorts, the signature seen was dominated by neutrophils, which formed the bulk of the circulating leukocytes. Multiple lymphocyte-related pathways were downregulated, but this reflects the fact that lymphocyte counts were low in both melioidosis and tuberculosis. Pathways associated with transcription and translation were also prominent, in keeping with the large number of genes differentially regulated in both melioidosis and tuberculosis.

**Table 1:** Patient characteristics.

	Melioidosis patients		
	No diabetes	Diabetes patients	
Parameter		On Gb	Not on Gb
Male gender	9 of 10	6 of 10	5 of 10
Age (years)	53	60	51
Glucose (mg dL^–1^)	128	218	256
Hb A_1c_ (%)	6.0	10.8	11.1
Neutrophils (×10^9^ L^–1^)	8.1	10.8	8.8
Lymphocytes (×10^9^ L^–1^)	1.0	1.6	1.5
Mortality	1 of 10	2 of 10	5 of 10^a^
	**Controls**		
	**No diabetes**	**Diabetes patients**	
**Parameter**		**On Gb**	**Not on Gb**
Male gender	10 of 10	3 of 10	5 of 10
Age (years)	40	54	56
Glucose (mg dL^–1^)	95	126	124
Neutrophils (×10^9^ L^–1^)	4.0	4.7	5.1
Lymphocytes (×10^9^ L^–1^)	1.9	4.4	5.1
Hb A_1c_ (%)	5.4	9.0	9.0

a One patient in this group was lost to follow-up following discharge from hospital, and was counted as having survived to discharge.

Gb = Glibenclamide. Values reported are means, except where stated.

### Pathway Analysis

**Figure 1:**
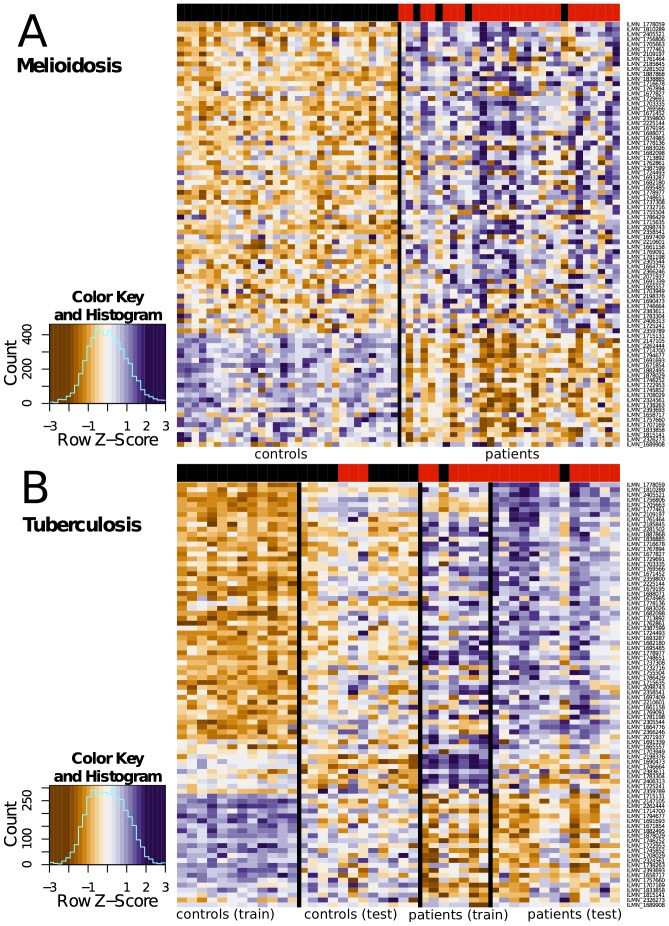
The 86-gene signature of tuberculosis is also seen in melioidosis. These heat maps demonstrate the gene expression profiles for two cohorts: (A) melioidosis and (B) tuberculosis. The 86 genes displayed are those identified by Berry *et al.* [7] as being specific for tuberculosis, after excluding genes differentially regulated in other infections (*Staphylococcus aureus* and Group A Streptococcus) and inflammatory conditions (adult onset Still’s disease and systemic lupus erythematosus). Each column in the heat map is the gene expression profile of an individual, with control subjects on the left and patients on the right. Each cell within the heat map is the expression of a single gene: orange genes are upregulated and purple genes are downregulated, with expression normalized across the rows. We used this 86-gene signature to cluster study participants into two groups (marked black and red in the coloured banner at the top of each heat map). In the tuberculosis cohort, three controls clustered with the patients, and two patients clustered with the controls. In the melioidosis cohort, the same 86-gene signature also allowed us to distinguish controls and patients, with the exception of four patients who clustered with the controls. Despite the same microarray platform being used, the two cohorts were assayed as separate batches, so the absolute fluorescence intensities are different, making a direct comparison of melioidosis and tuberculosis impossible. All patients were therefore compared to their own controls.

Interferon-mediated responses were the dominant pathway seen in both melioidosis and in tuberculosis (*p*<0.0001 for both, Tables 2 & 3). Class 1 and class 2 interferons were prominent in both (Table 4). Of the immune-related pathways, TRAIL (TNF superfamily member 10), tumour necrosis factor α (TNFα), transforming growth factor β (TGF-β), interleukin (IL)-1, IL-2, IL-12, chemokine and Toll-like receptor (TLR) pathways were all differentially regulated (Tables 2 & 3). There was no gene signature that distinguished melioidosis from tuberculosis, and for each of the pathways differentially expressed in melioidosis, we were able to find a counterpart in tuberculosis (Tables 2 & 3). Berry et al. reported an 86-gene signature that was specific for tuberculosis [7]. This 86-gene signature was also present in melioidosis (Figure 1).

**Table 2:** Genes upregulated in melioidosis and tuberculosis, arranged by pathway.

	Melioidosis (total = 651)			Tuberculosis (total = 847)		
Pathway	Genes	List	*p*-value	Genes	List	*p*-value
IFN-γ pathway	61	MEF2A, SLC9A1, IL4R, LYN, SLC3A2, TXN, PML, MKNK1, CDKN2B, RALB, IL1B, JUNB, DDIT3, COL1A2, FGR, TFE3, VDR, ATF6, CDKN1A, FES, MAX, CASP1, PPP2R2A, CEBPB, HCK, JAK2, JAK3, STAT5A, STAT5B, LAMC1, PPARG, PTEN, GADD45G, GADD45B, GADD45A, MAP3K11, FKBP1A, ZBTB17, BCL6, CSNK1A1, YWHAB, STAT1, MAPK13, MAPK14, SOCS3, SOCS1, GRB2, ITGB3, RUNX1, PTPN6, PTPN2, EIF4E, SAP30, IFNGR1, PRKCD, TNF, XIAP, MAP3K3, IL2RG, IRF7, IRF1	<0.0001	79	MEF2A, MAP3K7, MAP3K8, MAPK1, MAPK3, DUSP10, ARG1, LYN, SLC3A2, TXN, GSK3B, RALB, IL1B, ARRB2, ATF6, CDKN1A, MAX, CASP1, PKN1, CEBPB, PARP14, PPARG, PTEN, RBBP4, RAB5A, YWHAZ, FKBP1A, BCL6, CSNK1A1, YWHAB, YWHAH, DYNLRB1, GRB2, ITGB3, RAP1A, PRKCD, MAP3K3, CAMK2D, NUP153, IRF7, IRF1, CTNNB1, TGFBR2, PML, PPP2CA, SOS1, PPP2CB, DAB2, JUNB, DDIT3, FGR, VDR, TRAF6, ZFYVE16, NCOA2, PPM1A, NR3C1, NEDD4L, JAK2, LAMC1, CREBBP, RHEB, GADD45G, GADD45B, GADD45A, HBP1, STAT1, MAPK14, SOCS3, SOCS1, ELK4, PTPN2, SAP30, PAK2, IFNGR1, EGR3, EGR2, DAPK1, PPP1R15A	<0.0001
Glypican network	60	MEF2A, NFKBIB, NFKBIA, ARF1, SLC9A1, NOD2, LYN, SLC3A2, TXN, PML, MKNK1, DAPP1, CDKN2B, RALB, RELB, JUNB, DDIT3, COL1A2, ARFGAP1, FGR, TFE3, MDK, VDR, VAV1, ATF6, CDKN1A, CDC42, MAX, CASP9, PPP2R2A, REL, CEBPB, HCK, LAMC1, ASAP1, GOSR2, PPARG, NFKB2, PTEN, GADD45G, GADD45B, GADD45A, FKBP1A, ZBTB17, CSNK1A1, YWHAB, MAPK13, MAPK14, PAG1, ERC1, GRB2, RUNX1, PTPN6, EIF4E, SAP30, PRKCD, TNF, XIAP, MAP3K3, IRF7	<0.0001	87	APP, ARRB2, ASAP1, ATF6, BCL10, BCL3, BIRC2, CASP9, CDC42, CDKN1A, CEBPB, CLTA, CREBBP, CSNK1A1, CTNNB1, DAB2, DAPP1, DDIT3, DUSP10, DYNLRB1, EGR2, EGR3, ELK4, FBXW11, FGR, FKBP1A, FZD1, GADD45A, GADD45B, GADD45G, GOSR2, GRB2, GSK3B, HBP1, HSP90AA1, IRF7, JUNB, LAMC1, LYN, MACF1, MAP3K3, MAP3K7, MAP3K8, MAPK1, MAPK14, MAPK3, MAX, MEF2A, NCOA2, NEDD4L, NFKBIA, NFKBIB, NR3C1, NRAS, NUP153, PAG1, PAK2, PKN1, PML, PPARG, PPM1A, PPP1R15A, PPP2CA, PPP2CB, PRKCD, PTEN, PTPRC, RAB5A, RALB, RAP1A, RBBP4, RELB, RHEB, RIPK2, SAP30, SLC3A2, SOS1, TGFBR2, TNFAIP3, TRAF6, TXN, UBE2D3, VDR, YWHAB, YWHAH, YWHAZ, ZFYVE16	<0.0001
TRAIL (TNF superfamily,member 10) signalling	47	NFKBIB, NFKBIA, ARF1, ASAH1, NOD2, LYN, SLC3A2, MKNK1, DAPP1, RELB, FADD, JUNB, ARFGAP1, FGR, VAV1, CDKN1A, TNFSF10, CASP4, CASP9, CASP7, CASP1, LIMK1, REL, HCK, SMPD1, KRT18, LMNB1, ASAP1, GOSR2, PPARG, NFKB2, PTEN, FKBP1A, CSNK1A1, YWHAB, MAPK14, PAG1, MAP4K4, ERC1, GRB2, PTPN6, EIF4E, PRKCD, CTSD, TNF, XIAP, CASP10	<0.0001	70	MAP3K7, APP, MAP3K8, MAPK1, MAPK3, NFKBIB, NFKBIA, ASAH1, LYN, AIFM1, SLC3A2, NRAS, GSK3B, BCL10, RELB, ARRB2, EGF, CDKN1A, CASP4, CASP9, CASP7, CASP8, CASP1, CASP2, CYCS, NSMAF, LMNB1, GOSR2, PPARG, PTEN, BID, YWHAZ, FKBP1A, BCL3, CSNK1A1, YWHAB, BIRC2, YWHAH, PDGFA, GRB2, UBE2D3, RAP1A, CLTA, PRKCD, DAPP1, SOS1, FBXW11, CRADD, JUNB, TNFAIP3, FGR, TRAF6, TNFSF10, LIMK1, RB1, VIM, SMPD1, ASAP1, CREBBP, RHEB, TNFRSF10C, MAPK14, PAG1, RIPK2, EGR3, HSP90AA1, EGR2, CTSD, PTPRC, CASP10	<0.0001
Plasma membrane estrogen receptor signalling	45	MEF2A, ROCK2, CTNNA1, PLAUR, GRB10, ARF1, SLC9A1, LNPEP, LYN, SLC3A2, MKNK1, EXOC6, EXOC5, TRIP10, GNA13, GNA15, RALB, JUNB, DDIT3, VAMP3, ARFGAP1, FGR, ATF6, RHOQ, SH2B2, CEBPB, HCK, ACAP1, ITGA2B, ASAP1, PXN, GOSR2, PPARG, FKBP1A, CSNK1A1, YWHAB, MAPK14, MMP9, GRB2, ITGB3, EIF4E, PRKCD, TNF, MAP3K3, CRK	<0.0001	54	MEF2A, MAP3K8, MAPK1, MAPK3, DUSP10, LNPEP, LYN, SLC3A2, EXOC7, EXOC6, EXOC1, NRAS, GSK3B, GNA13, RALB, ARRB2, VAMP3, EGF, ATF6, CEBPB, ACAP2, GOSR2, PPARG, RAB5A, YWHAZ, FKBP1A, CSNK1A1, YWHAB, YWHAH, GRB2, ITGB3, CLTA, PRKCD, MAP3K3, CTNNB1, CTNNA1, PLAUR, GNAI3, GNAQ, SOS1, RAB11A, JUNB, DDIT3, FGR, TRAF6, ASAP1, CREBBP, HBP1, MAPK14, ELK4, PAK2, EGR3, EGR2, PTPRA	<0.0001
TNF-α/NF-κB signalling	44	MEF2A, NFKBIA, SLC9A1, ASAH1, NOD2, LYN, SLC3A2, TXN, MKNK1, RALB, RELB, FADD, JUNB, DDIT3, FGR, ATF6, CASP4, CASP9, CASP7, CASP1, LIMK1, REL, CEBPB, HCK, SMPD1, KRT18, LMNB1, PPARG, NFKB2, ADAM17, FKBP1A, CSNK1A1, YWHAB, STAT1, MAPK14, MAP4K4, ERC1, EIF4E, PRKCD, CTSD, TNF, XIAP, MAP3K3, CASP10	<0.0001	68	MEF2A, MAP3K7, APP, MAP3K8, MAPK1, MAPK3, DUSP10, NFKBIA, ASAH1, LYN, AIFM1, SLC3A2, TXN, GSK3B, RALB, BCL10, RELB, ARRB2, EGF, ATF6, CASP4, CASP9, CASP7, CASP8, CASP1, CASP2, CYCS, CEBPB, NSMAF, LMNB1, PPARG, RAB5A, BID, YWHAZ, FKBP1A, BCL3, CSNK1A1, YWHAB, BIRC2, YWHAH, PDGFA, UBE2D3, PRKCD, MAP3K3, FBXW11, CRADD, JUNB, DDIT3, TNFAIP3, FGR, TRAF6, LIMK1, RB1, VIM, SMPD1, CREBBP, ADAM17, HBP1, STAT1, MAPK14, MAP4K3, RIPK2, ELK4, PAK2, EGR3, EGR2, CTSD, CASP10	<0.0001
TGF-β receptor signalling; regulation of cytoplasmic and nuclear SMAD2/3 signalling	41	MEF2A, SLC9A1, LYN, SLC3A2, TXN, PML, MKNK1, CDKN2B, RALB, JUNB, DDIT3, COL1A2, FGR, TFE3, VDR, ATF6, CDKN1A, MAX, PPP2R2A, CEBPB, HCK, LAMC1, PPARG, GADD45G, GADD45B, GADD45A, FKBP1A, ZBTB17, CSNK1A1, YWHAB, MAPK13, MAPK14, GRB2, RUNX1, EIF4E, SAP30, PRKCD, TNF, XIAP, MAP3K3, IRF7	<0.0001	61	MEF2A, MAP3K7, MAP3K8, MAPK1, MAPK3, DUSP10, LYN, SLC3A2, TXN, GSK3B, RALB, ARRB2, ATF6, CDKN1A, MAX, PKN1, CEBPB, PPARG, RBBP4, RAB5A, YWHAZ, FKBP1A, CSNK1A1, YWHAB, YWHAH, DYNLRB1, GRB2, PRKCD, MAP3K3, NUP153, IRF7, CTNNB1, TGFBR2, PML, PPP2CA, SOS1, PPP2CB, DAB2, JUNB, DDIT3, FGR, VDR, TRAF6, ZFYVE16, NCOA2, PPM1A, NR3C1, NEDD4L, LAMC1, CREBBP, GADD45G, GADD45B, GADD45A, HBP1, MAPK14, ELK4, SAP30, PAK2, EGR3, EGR2, PPP1R15A	<0.0001
IL-1-mediated signalling	35	MEF2A, IL1R2, NFKBIA, IL1RN, SLC9A1, NOD2, LYN, SLC3A2, MKNK1, RALB, IL1B, RELB, JUNB, DDIT3, FGR, ATF6, CASP1, REL, CEBPB, HCK, MYD88, TOLLIP, PPARG, NFKB2, FKBP1A, CSNK1A1, YWHAB, MAPK14, ERC1, IRAK4, IRAK3, EIF4E, PRKCD, TNF, MAP3K3	<0.0001	48	MEF2A, MAP3K7, MAP3K8, MAPK3, DUSP10, NFKBIA, LYN, SLC3A2, GSK3B, RALB, IL1B, BCL10, RELB, ARRB2, UBE2V1, ATF6, CASP1, CEBPB, MYD88, PPARG, RAB5A, YWHAZ, FKBP1A, BCL3, CSNK1A1, YWHAB, BIRC2, YWHAH, IRAK4, IRAK3, UBE2D3, PRKCD, MAP3K3, IL1RN, FBXW11, JUNB, DDIT3, TNFAIP3, FGR, TRAF6, CREBBP, HBP1, MAPK14, RIPK2, ELK4, PAK2, EGR3, EGR2	<0.0001
Chemokine signalling	29	GNG8, GNG5, PIK3CG, ROCK2, NFKBIB, NFKBIA, LYN, GNB4, PTK2B, SOS2, CXCL10, FGR, VAV1, CXCL16, CDC42, HCK, JAK2, JAK3, ADCY3, ADCY4, STAT5B, CCR2, PXN, CCR1, STAT1, STAT3, GRB2, PRKCD, CRK	0.0001	37	PIK3CB, MAPK1, MAPK3, NFKBIB, NFKBIA, LYN, NRAS, GSK3B, CXCL10, ARRB2, CXCL16, CCR2, CXCL5, CCR1, GRB2, RAP1A, PRKCD, GNG5, ROCK1, NCF1, GNAI3, GNB4, GNG11, CCL28, PTK2B, SOS1, SOS2, GNG10, FGR, VAV3, CDC42, JAK2, ADCY7, ADRBK2, STAT1, STAT3, STAT2	<0.0001
p75 (NTR) signalling	28	BCL2L11, NFKBIA, NOD2, SORT1, RELB, CDC42, CASP9, CDK5, APH1B, REL, NCSTN, STAT5A, MYD88, TRPC3, RIT1, NFKB2, ADAM17, YWHAB, STAT3, MAPK14, ERC1, GRB2, DYNLT1, PRKCD, TNF, XIAP, RGS19, CRK	0.0001	40	APP, MAPK1, MAPK3, NFKBIA, SORT1, NRAS, GSK3B, FRS3, BCL10, RELB, ARRB2, BEX1, CASP9, CYCS, MYD88, RIT1, YWHAZ, BCL3, YWHAB, BIRC2, YWHAH, GRB2, UBE2D3, RAP1A, PRKCD, SOS1, FBXW11, TNFAIP3, TRAF6, CDC42, APH1B, NEDD4L, GAB2, NGFRAP1, PSEN1, ADAM17, MAPK10, STAT3, MAPK14, RIPK2	<0.0001
Phagosome	28	NCF4, MSR1, HLA-B, HLA-G, VAMP3, STX7, ATP6V1H, NOX3, CYBA, CYBB, CD36, TUBA4A, CTSL1, ATP6V0E1, FCGR1A, THBS1, ATP6V1E1, FCGR2A, CD14, TLR2, TLR4, TLR6, ITGB2, ITGB3, ITGAM, FCAR, TAP2, TAP1	<0.0001	41	HLA-DRA, HLA-B, HLA-F, VAMP3, RAB7A, HLA-DMB, CYBB, CD36, STX12, ATP6V0E1, RAB5C, FCGR3A, FCGR3B, RAB5A, FCGR1A, TUBA8, FCGR2A, CD14, ITGB3, FCAR, TUBA1A, TUBA1B, TAP2, TAP1, NCF2, NCF1, NCF4, HLA-DPA1, HLA-DPB1, MSR1, EEA1, STX7, TCIRG1, CTSL1, DYNC1I2, ATP6V1G1, ATP6V1E1, TLR2, TLR4, TLR6, ACTB	<0.0001
Apoptosis	26	H1F0, BCL2L11, FADD, TNFSF10, CASP9, CASP7, LMNB1, YWHAB, PSMD12, TJP2, PSMA6, PSMA4, PSMA3, PSMB7, PSMB3, PSMB2, BMX, PSMB8, PSMB9, PSMC1, PSMD6, PSMD9, PRKCD, TNF, XIAP, CASP10	<0.0001	21	PIK3CB, NFKBIA, AIFM1, IL1B, CASP9, CASP7, CASP8, CYCS, MYD88, BID, BIRC2, IRAK4, IRAK3, IRAK2, FAS, TNFSF10, PRKAR1A, CSF2RB, TNFRSF10C, PPP3R1, CASP10	<0.0001
Toll-like receptor signalling	25	PIK3CG, NFKBIA, CXCL10, IL1B, FADD, TRAF3, MYD88, TOLLIP, LY96, STAT1, MAPK13, MAPK14, CD14, TBK1, TLR1, TLR2, TLR4, TLR5, TLR6, TLR8, IRAK4, IFNAR1, IFNAR2, TNF, IRF7	<0.0001	29	MAP3K7, MAP3K8, PIK3CB, MAPK1, MAPK3, NFKBIA, CXCL10, IL1B, CASP8, MYD88, CD40, LY96, CD14, IRAK4, CD86, IRF7, TRAF6, MAPK10, STAT1, MAPK14, TBK1, TLR2, TLR4, TLR5, TLR6, TLR7, TLR8, IFNAR1, IFNAR2	<0.0001
IL-12 mediated signalling events	21	NFKBIA, NOD2, IL1B, IL27, RELB, IL18RAP, REL, JAK2, STAT5A, NFKB2, GADD45G, GADD45B, IL18R1, STAT1, STAT3, MAPK14, SOCS1, ERC1, TNF, IL2RG, IRF1	0.0001	31	HLA-DRA, NFKBIA, IL1B, BCL10, IL27, RELB, ARRB2, RAB7A, IL12RB1, IL18RAP, IL6ST, PIAS2, BCL3, BIRC2, UBE2D3, CD86, IRF1, FBXW11, TNFAIP3, TRAF6, JAK2, CREBBP, GADD45G, GADD45B, IL18R1, STAT1, STAT3, STAT2, MAPK14, SOCS1, RIPK2	<0.0001
IL-2 mediated signalling	20	UGCG, NFKBIA, NOD2, PTK2B, RELB, REL, SMPD1, JAK3, STAT5A, STAT5B, NFKB2, STAT1, STAT3, MAPK14, SOCS3, SOCS1, ERC1, GRB2, TNF, IL2RG	0.0002	31	SGMS1, UGCG, MAPK1, MAPK3, NFKBIA, ELF1, NRAS, BCL10, RELB, ARRB2, STAM2, BCL3, BIRC2, GRB2, UBE2D3, TERT, CCNA2, PTK2B, SOS1, FBXW11, TNFAIP3, TRAF6, SMPD1, GAB2, STAT1, STAT3, MAPK14, SOCS3, SOCS1, RIPK2, HSP90AA1	<0.0001
NOD-like receptor signalling	15	NFKBIB, NFKBIA, NOD2, NLRC4, IL1B, TRIP6, CASP5, CASP1, MEFV, ERBB2IP, MAPK13, MAPK14, TNF, XIAP, PYCARD	0.0001	21	MAP3K7, MAPK1, MAPK3, NFKBIB, NFKBIA, NLRC4, IL1B, CASP5, CASP8, CASP1, BIRC2, CARD6, PYCARD, TNFAIP3, TRAF6, MEFV, MAPK10, ERBB2IP, MAPK14, RIPK2, HSP90AA1	<0.0001

**Table 3:** Genes downregulated in melioidosis and tuberculosis, arranged by pathway.

	Melioidosis (total = 1007)			Tuberculosis (total = 1138)		
GeneSet	Genes	List	*p*-value	Genes	List	*p*-value
Glypican network	99	MEF2C, MAP3K4, PPP1CA, ZAP70, ATM, CARD11, PPP3CC, IL8, MEF2D, RALA, ATF2, CCM2, CDKN1B, HDAC1, CD4, NFATC3, MAF, CABIN1, WWP1, CAMK4, ETS1, AES, CTBP1, RBBP7, MAP3K14, BCL2, CD22, FYN, YWHAQ, FOXO1, FOXO4, GATA3, BLK, EIF4B, SPTBN1, CD79B, CD79A, PLEKHA2, PLEKHA1, CLTB, RANBP3, PRKCH, LEF1, UBE2I, PRKCQ, PRNP, CYTH1, CAMK2G, CYTH3, MAP3K3, PIK3R1, PTGS2, CTGF, GNG2, TGIF1, TGIF2, TCF3, RPS6, TGFBR3, MAPT, MYC, ARHGEF7, GNB1, DGKA, TBC1D4, CTLA4, RPS6KA5, PDE3B, CTDSP2, EEF2, LAT, TSC1, SGK1, LCK, NR3C1, HSPA8, CREB1, CDC25B, SNIP1, SIN3A, CD40LG, SMAD4, SMAD3, CBLB, CYLD, STRAP, MAP4K1, SRF, AXIN2, RUNX2, RUNX3, AXIN1, ITK, PTPN6, MALT1, PLCG1, AKT1, GSC, PTPRC	<0.0001	105	HRAS, PPP2R5D, MAP3K7, MAP3K4, ZAP70, ATM, EEF2K, CARD11, PPP3CB, PPP3CC, IL4, MEF2D, DUSP8, IL10, RALA, ARFGAP1, MAPKAPK5, HDAC2, CDKN1B, HDAC1, IKBKB, PPP2R2A, CD4, NFATC3, MAF, CABIN1, WWP1, TGFBRAP1, CD72, ETS1, AES, CTBP1, RBBP4, RBBP7, MAP3K14, BCL2, CD22, CD19, FYN, FOXO1, FOXO3, GATA3, BLK, EIF3A, CD79B, CD79A, SHC1, PRKCA, LEF1, PRKCH, UBE2I, PRKCQ, CYTH1, CAMK2G, MAP3K1, YES1, IRF4, IBTK, TGFBR2, RPS6, TGFBR3, MYC, ARHGEF7, TLE1, GNB1, TBC1D4, CTLA4, RPS6KA5, XPO1, RPS6KB1, CTDSP1, CTDSP2, EEF2, TRAF2, LAT, TSC1, TSC2, SGK1, CDK4, LCK, INPP5D, HSPA8, CREB1, SIN3B, SIN3A, CD40LG, MACF1, SMAD7, SMAD3, DCP1A, CYLD, FZD3, MAPK13, CALM1, MAP4K1, AXIN2, RUNX2, RUNX3, AXIN1, ITK, MALT1, PLCG1, AKT1, PAK2, CSK	<0.0001
Protein synthesis, RNA translation, ribosome	85	RPLP2, RPLP0, RPLP1, PABPC1, EIF4A2, EEF1G, EEF1D, RPS27A, RPL35A, RPL10A, DOHH, RPL37A, RPL13A, CCT3, CCT7, RPL18A, EIF5A, EEF1B2, EIF3D, EIF3B, EIF3G, EIF3H, EIF3E, EIF3F, EIF3K, EEF1A1, EIF4B, EIF4H, DHPS, GSPT2, RPL18, RPL17, RPL19, RPL14, RPL13, RPL15, RPL10, RPL11, RPL12, EIF2S3, RPS18, RPS19, RPS16, RPS14, RPS15, RPS12, RPS13, RPS10, RPS11, RPS25, RPS27, RPS28, RPS29, RPS20, RPS23, RPS6, RPS5, RPL35, RPL36, RPL38, RPL30, RPL32, RPL27, RPL29, RPL22, DPM2, EIF5, EEF2, UBA52, RPL7, RPL6, RPL8, RPL3, RPL5, RPL4, RPL7A, RPL23A, RPS2, RPS3, RPS4X, RPS15A, RPL27A, EIF2B1, PIGA, PIGP	<0.0001	80	CCT2, CCT3, CCT6A, CCT7, EEF1B2, EEF1D, EEF1G, EEF2, EIF1AX, EIF2B1, EIF2B4, EIF2B5, EIF2S3, EIF3A, EIF3B, EIF3D, EIF3F, EIF3G, EIF3H, EIF3I, EIF3K, EIF4A2, FBXW7, PABPC1, RPL10, RPL10A, RPL11, RPL13, RPL13A, RPL14, RPL15, RPL18, RPL19, RPL22, RPL23, RPL23A, RPL26, RPL27, RPL29, RPL3, RPL31, RPL32, RPL35, RPL36, RPL36A, RPL37A, RPL4, RPL5, RPL6, RPL7, RPL7A, RPL8, RPL9, RPLP0, RPLP1, RPLP2, RPS11, RPS13, RPS15, RPS15A, RPS16, RPS18, RPS19, RPS2, RPS20, RPS23, RPS24, RPS25, RPS27, RPS27A, RPS28, RPS29, RPS3, RPS3A, RPS4X, RPS5, RPS6, RPS7, RPS9, UBA52	<0.0001
TGF-β receptor signalling,regulation of SMAD2/3	76	MEF2C, MAP3K4, PPP1CA, ATM, IL8, MEF2D, RALA, ATF2, CCM2, HDAC1, NFATC3, MAF, CABIN1, WWP1, CAMK4, CTBP1, RBBP7, BCL2, FYN, YWHAQ, FOXO1, FOXO4, GATA3, BLK, SPTBN1, PRKCH, UBE2I, PRKCQ, MAP3K3, PTGS2, CTGF, TGIF1, TGIF2, TCF3, TGFBR3, MAPT, MYC, DGKA, CTLA4, RPS6KA5, CTDSP2, LCK, NR3C1, HSPA8, CREB1, CDC25B, SNIP1, SIN3A, CD40LG, SMAD4, SMAD3, CBLB, STRAP, SRF, RUNX2, RUNX3, AXIN1, GSC, TGFB3, FNTA, E2F4, ANAPC1, ANAPC5, ETS1, SNW1, LEF1, CAMK2G, PIK3R1, PIK3R2, FOSB, RBL2, JUND, CTCF, CDC23, GIPC1, AXIN2	<0.0001	61	MAP3K7, MAP3K4, ATM, EEF2K, IL4, MEF2D, DUSP8, IL10, RALA, MAPKAPK5, HDAC2, HDAC1, PPP2R2A, NFATC3, MAF, CABIN1, WWP1, TGFBRAP1, CTBP1, RBBP4, RBBP7, BCL2, FYN, FOXO1, FOXO3, GATA3, BLK, SHC1, PRKCA, PRKCH, UBE2I, PRKCQ, MAP3K1, YES1, IRF4, TGFBR2, TGFBR3, MYC, CTLA4, RPS6KA5, RPS6KB1, CTDSP1, CTDSP2, TRAF2, TSC2, CDK4, LCK, HSPA8, CREB1, SIN3B, SIN3A, CD40LG, SMAD7, SMAD3, DCP1A, MAPK13, CALM1, RUNX2, RUNX3, AXIN1, PAK2	<0.0001
IFN-γ pathway	72	MEF2C, MAP3K4, PPP1CA, ATM, DOK2, IL8, MEF2D, RALA, ATF2, CCM2, HDAC1, NFATC3, MAF, CABIN1, WWP1, CAMK4, ETS1, CTBP1, RBBP7, BCL2, FYN, YWHAQ, FOXO1, FOXO4, GATA3, BLK, EIF4B, SPTBN1, PRKCH, UBE2I, PRKCQ, CAMK2G, MAP3K3, PIK3R1, PTGS2, CTGF, TGIF1, TGIF2, TCF3, RPS6, TGFBR3, MAPT, MYC, DGKA, CTLA4, RPS6KA5, CTDSP2, EEF2, TSC1, LCK, NR3C1, HSPA8, CREB1, CDC25B, JAK1, SNIP1, SIN3A, RAPGEF1, CD40LG, FCER2, SMAD4, SMAD3, PTPN11, CBLB, STRAP, SRF, RUNX2, RUNX3, AXIN1, PTPN6, AKT1, GSC	<0.0001	82	PPP2R5D, MAP3K7, MAP3K4, ATM, EEF2K, DOK2, IL4, MEF2D, DUSP8, IL10, RALA, MAPKAPK5, TFF3, HDAC2, HDAC1, PPP2R2A, NFATC3, MAF, CABIN1, WWP1, TGFBRAP1, PIAS1, ETS1, CTBP1, RBBP4, RBBP7, MAP3K11, BCL2, FYN, FOXO1, FOXO3, GATA3, BLK, EIF3A, SHC1, PRKCA, PRKCH, UBE2I, PRKCQ, CAMK2G, MAP3K1, YES1, IRF4, TGFBR2, RPS6, TGFBR3, MYB, MYC, CTLA4, RPS6KA5, RPS6KB1, CTDSP1, CTDSP2, EEF2, TRAF2, TSC1, TSC2, CDK4, HMGA1, LCK, INPP5D, HSPA8, CREB1, SIN3B, SIN3A, RAPGEF1, CD40LG, FCER2, SMAD7, SMAD3, PTPN11, DCP1A, GTF3A, STAT6, MAPK13, CALM1, RUNX2, RUNX3, AXIN1, AKT1, PAK2, LTA	<0.0001
TRAIL (TNF superfamily, member 10) signalling	70	ZAP70, ATM, PARP1, CARD11, PPP3CC, IL8, LMNA, MEF2D, CDKN1B, CASP8, CASP2, CYCS, CD4, NFATC3, MAF, CABIN1, CAMK4, ETS1, MAP3K14, PRF1, BCL2, CD22, FYN, YWHAQ, FOXO1, FOXO4, GATA3, BLK, EIF4B, CD79B, CD79A, PLEKHA2, PLEKHA1, CLTB, PRKCH, PRKCQ, CFL2, CYTH1, CAMK2G, CYTH3, PIK3R1, PTGS2, RPS6, MYC, ARHGEF7, DGKA, NUMA1, TBC1D4, CTLA4, PDE3B, EEF2, TRADD, LAT, TSC1, SGK1, LCK, CD40LG, CBLB, CYLD, SATB1, MADD, MAP4K1, ITK, PTPN6, MALT1, PLCG1, DAP3, AKT1, PTPRC, SPTAN1	<0.0001	83	HRAS, PPP2R5D, MAP3K7, ZAP70, ATM, PARP1, AIFM1, CARD11, PPP3CB, PPP3CC, IL4, MEF2D, DFFA, DFFB, TFAP2A, ARFGAP1, CDKN1B, IKBKB, LRDD, CYCS, CD4, NFATC3, MAF, CABIN1, KRT18, CD72, ETS1, MAP3K14, PRF1, BCL2, CD22, BIRC3, CD19, FYN, FOXO1, FOXO3, GATA3, BLK, EIF3A, CD79B, CD79A, SHC1, PRKCA, PRKCH, PRKCQ, CYTH1, CAMK2G, MAP3K1, YES1, IRF4, IBTK, RPS6, MYC, ARHGEF7, NUMA1, TBC1D4, CTLA4, XPO1, RPS6KB1, EEF2, TRAF2, TRADD, LAT, TSC1, TSC2, SGK1, CDK4, LCK, INPP5D, SMPD1, CD40LG, TNFRSF10B, CYLD, CALM1, SATB1, MAP4K1, ITK, MALT1, PLCG1, DAP3, AKT1, CSK, SPTAN1	<0.0001
Class I PI3K signalling events	56	ZAP70, ATM, CARD11, PPP3CC, IL8, MEF2D, CDKN1B, CD4, NFATC3, MAF, CABIN1, CAMK4, ETS1, MAP3K14, BCL2, CD22, FYN, YWHAQ, FOXO1, FOXO4, GATA3, BLK, EIF4B, CD79B, CD79A, PLEKHA2, PLEKHA1, CLTB, PRKCH, PRKCQ, CYTH1, CAMK2G, CYTH3, PIK3R1, PTGS2, RPS6, ARHGEF7, DGKA, TBC1D4, CTLA4, PDE3B, EEF2, LAT, TSC1, SGK1, LCK, CD40LG, CBLB, CYLD, MAP4K1, ITK, PTPN6, MALT1, PLCG1, AKT1, PTPRC	<0.0001	64	HRAS, PPP2R5D, MAP3K7, ZAP70, ATM, CARD11, PPP3CB, PPP3CC, IL4, MEF2D, ARFGAP1, CDKN1B, IKBKB, CD4, NFATC3, MAF, CABIN1, CD72, ETS1, MAP3K14, BCL2, CD22, CD19, FYN, FOXO1, FOXO3, GATA3, BLK, EIF3A, CD79B, CD79A, SHC1, PRKCA, PRKCH, PRKCQ, CYTH1, CAMK2G, MAP3K1, YES1, IRF4, IBTK, RPS6, ARHGEF7, TBC1D4, CTLA4, XPO1, RPS6KB1, EEF2, LAT, TSC1, TSC2, SGK1, CDK4, LCK, INPP5D, CD40LG, CYLD, CALM1, MAP4K1, ITK, MALT1, PLCG1, AKT1, CSK	<0.0001
TNF- α/NF-κB	51	MEF2C, ATM, PARP1, IL8, LMNA, MEF2D, RALA, ATF2, CCM2, CASP8, CASP2, CYCS, NFATC3, MAF, CABIN1, CAMK4, GNB2L1, MAP3K14, PRF1, BCL2, FYN, YWHAQ, GATA3, BLK, PRKCH, PRKCQ, CFL2, MAP3K3, PIK3R1, PTGS2, TCF3, MYC, DGKA, NUMA1, CTLA4, RPS6KA5, TRADD, LCK, CREB1, CDC25B, CD40LG, CBLB, CYLD, SATB1, MADD, MAP4K5, MAP4K2, SRF, MALT1, AKT1, SPTAN1	<0.0001	34	SMARCA4, SMARCB1, IKBKE, HDAC2, HDAC1, IKBKB, FAF1, MAP3K14, BIRC3, AKAP8, MAP3K2, MAP2K5, RPS13, BTRC, RPS6KB1, LRPPRC, TRAF1, TRAF2, RPL6, RPL4, TRAF5, TRADD, FBL, PTPN11, POLR1C, POLR2H, HSP90AB1, AKT1, MCM7, PSMD1, MCM5, KPNA6, PEBP1, KPNA3	<0.0001
Formation and maturation of mRNA transcript	47	PTBP1, DHX9, YBX1, SF3B4, SF3B3, SF3A2, SF3A1, SF3A3, HNRNPUL1, TXNL4A, NCBP2, SNRPD3, DNAJC8, PRPF6, SNRNP200, PRPF8, HNRNPA0, TH1L, PCBP1, PCBP2, ERCC3, LSM2, NHP2L1, GTF2H3, TCEA1, RNMT, RBM5, SNRPA1, HNRNPR, HNRNPU, HNRNPM, HNRNPK, HNRNPD, HNRNPC, TAF4, RBMX, HNRNPH1, CPSF1, U2AF2, CCNT2, SNRNP40, POLR2H, POLR2G, SNRNP70, SRRM1, SNRPB, RNPS1	<0.0001	49	TAF4B, PTBP1, SF3B4, SF3B3, SF3B1, SF3A2, SF3A1, SF3A3, HNRNPUL1, NCBP2, DNAJC8, PRPF4, SNRNP200, PRPF8, HNRNPA1, SUPT16H, TH1L, ERCC3, ERCC2, LSM2, NHP2L1, GTF2H1, RNMT, RBM5, DDX23, SNRPA1, HNRNPU, HNRNPM, METTL3, HNRNPD, TAF4, TAF6, HNRNPH1, COBRA1, CPSF1, SUPT5H, RNGTT, POLR2H, POLR2G, POLR2F, POLR2I, POLR2A, SNRNP70, SRRM1, EFTUD2, SNRPB, SNRPA, RNPS1, SMC1A	<0.0001
Spliceosome	37	THOC3, THOC1, SF3B4, SF3B3, SF3A2, SF3A1, SF3A3, TXNL4A, NCBP2, SNRPD3, PRPF3, PRPF6, SNRNP200, PRPF8, DDX5, SNW1, PPIE, PUF60, PCBP1, LSM4, LSM2, NHP2L1, SNRPA1, HNRNPU, HNRNPM, HNRNPK, TCERG1, DDX46, HNRNPC, RBMX, WBP11, HSPA8, U2AF2, SNRNP40, SNRNP70, RBM17, SNRPB	<0.0001	39	XAB2, THOC2, THOC1, SF3B4, SF3B3, SF3B1, DHX15, DHX16, SF3A2, SF3A1, SF3A3, NCBP2, PRPF3, PRPF4, SNRNP200, PRPF8, DDX5, HNRNPA1, PPIH, PUF60, LSM7, LSM5, LSM4, LSM2, NHP2L1, HSPA1A, DDX23, SNRPA1, HNRNPU, HNRNPM, DDX42, HSPA8, SNRNP70, RBM25, RBM17, EFTUD2, SNRPB, SNRPA, SNRPC	<0.0001
T-cell receptor signalling	34	ZAP70, CARD11, PPP3CC, ICOS, CD4, NFATC3, CD3G, CD3D, CD3E, MAP3K14, CD28, FYN, CD8A, CD8B, PRKCQ, PIK3R1, PIK3R2, CTLA4, LAT, LCK, CD40LG, CBLB, ITK, PTPN6, MALT1, PLCG1, AKT1, PAK4, CD247, PTPRC, HLA-DOA, HLA-DOB, RPS27A, HLA-DPA1	<0.0001	38	HRAS, MAP3K7, ZAP70, ATM, CARD11, IL4, IKBKB, CD4, NFATC3, CABIN1, CD3D, CD3E, MAP3K14, CD28, FYN, SHC1, PRKCA, PRKCQ, MAP3K1, XPO1, STK39, LAT, LCK, INPP5D, CD40LG, PTPN11, CYLD, CALM1, MAP4K1, PTPN7, ITK, MALT1, PLCG1, AKT1, CSK, CD247, RASGRP1, RASGRP2	<0.0001
IL-2-mediated signalling events	18	ATM, DOK2, IKZF3, MAP3K14, PRF1, BCL2, FYN, PIK3R1, IL2RB, RPS6, MYC, CCND2, LCK, JAK1, PTPN11, CYLD, MALT1, AKT1	0.022	30	HRAS, ATM, DOK2, IL4, IKBKB, MAP3K14, PRF1, BCL2, FYN, FOXO3, EIF3A, SHC1, PRKCA, IL2RB, RPS6, MYB, MYC, CCND2, XPO1, RPS6KB1, CDK6, LCK, SMPD1, PTPN11, CYLD, CISH, CALM1, MALT1, AKT1, LTA	<0.0001

IFN = interferon, IGF = insulin-like growth factor, IL = interleukin, NF-κB = nuclear factor kappa-light-chain-enhancer of activated B cells, PI3K = phosphoinositide 3-kinase, RNA = ribonucleic acid, TGF = transforming growth factor, TNF = tumour necrosis factor.

Note:– Genes names are those assigned by the HUGO gene nomenclature committee.

**Table 4:** Interferon signatures for melioidosis and tuberculosis.

Melioidosis			Tuberculosis		
*Type 1*	*Both*	*Type 2*	*Type 1*	*Both*	*Type 2*
AIM2CTSASH3GLB1TCN2NEU1FCGR1AH2AFJIFITM3LACTBDYSFVNN1PGS1CXCL16	TLR5GYG1DUSP3CASP1IFITM1UPP1SERPING1VAMP5SOCS3SAT1SLC30A1JAG1LIMK2DYNLT1TXNMYD88TAP1JAK2IL15	TNFSF10DRAMNEUR1CASP5SEPT4CD274SYN2H4HIST2H4BRAB24	GBP5ANKRD22FCGR1AAIM2CEACAM1WDFY1EPSTI1LY96GADD45BLACTBSP140SERTAD3SH3GLB1	CASP1DUSP3GBP1GBP2GBP4IL15ATF3PSME1PSME2UBE2L6GSTO1GCH1TAP1TRIM22STAT1TAP2PSMB8VAMP5WARSSECTM1GYG1SERPING1IRF1SAT1RTP4CLIC1CASP4PLAURDYNLT1SLC30A1ACTA2	SEPT4CD274NLRC5PSMB10P2RY14RNF213

Note: The interferon signatures for melioidosis (**A**) and tuberculosis (**B**) are listed here (analysis from www.interferome.org). Berry *et al.* noted that both type 1 and type 2 interferon responses were prominent in tuberculosis. We find that type 1 interferon responses appear in melioidosis also.

### Modular Analysis

**Figure 2:**
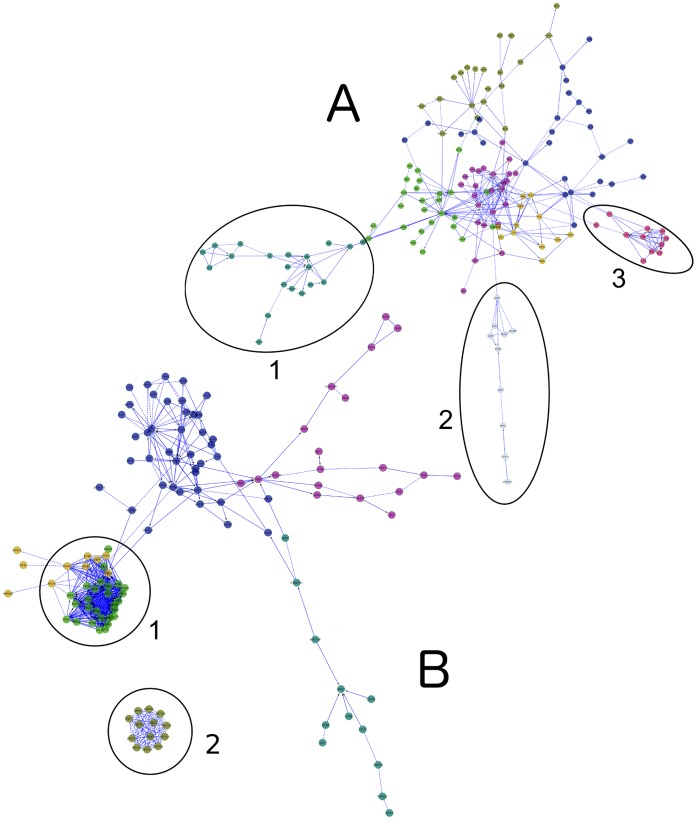
Network representation of genes differentially expressed in melioidosis. ‘Canonical’ pathways (such as those presented in a standard biochemistry textbook) are manually curated collections of protein interactions arranged in a manner that aids human understanding, and as artificial constructs the boundaries between pathways are subjective. Pathways that are conceptually distinct often have proteins in common and overlap, so in modular analysis, multiple pathways may collapse into a single module, causing other pathways and relationships to gain prominence. These two networks (**A** and **B**) represent those genes that are differentially expressed in melioidosis. For simplicity of presentation, we have used only a subset of genes in these networks. The top 221 upregulated genes (as ranked by *p*-value) are presented in **A**, and the top 155 downregulated genes are in **B**. The same clusters were found in an analysis of the whole gene set and those results are presented in Tables 2 & 3. **Network** **A**. IFN-γ, TNF-α, IL-12 signalling pathways cluster together with the glypican network in the centre of the graph, but the complement/chemokine receptor (**cluster 1**), inflammasome (**cluster 2**) and Toll-like receptor pathways come to prominence in this analysis (**cluster 3**). **Network B**. IFN-γ, TGF-β and TNF signalling again cluster in the middle of the network. The two most prominent clusters are ribosomal proteins (**cluster 1**) and zinc finger proteins (**cluster 2**).

In a modular analysis of the upregulated genes (Figure 2A), interferon and cytokine signalling clustered together in the centre of the network, causing the complement (cluster 1), NOD-like receptor (cluster 2) and TLR (cluster 3) pathways to gain prominence. In the downregulated genes (Figure 2B), the most prominent clusters were the ribosomal proteins (cluster 1) and zinc finger proteins (cluster 2).

### PAMP-specific Responses

*B. pseudomallei* expresses lipopolysaccharide on its outer membrane, while *M. tuberculosis* does not and has a lipid-rich cell wall. Lipopolysaccharide is recognized by TLR4 and CD14, and both are upregulated in melioidosis (*P* = 0.0016 and 1.5 × 10^–6^, respectively); however, TLR4 and CD14 are also upregulated in tuberculosis (*P* = 1.5 × 10^–6^ and 9.4 × 10^–4^). *B. pseudomallei* is a flagellated, motile bacterium, while *M. tuberculosis* is immotile with no flagellum. Flagellin is a ligand for TLR5 [25] and NLRC4 [26]. Both TLR5 and NLRC4 were upregulated in melioidosis (*P* = 5.4 × 10^–13^ and 4.2 × 10^–10^, respectively), but both were upregulated in tuberculosis also (*P* = 8.1 × 10^–10^ and 2.4 × 10^–11^).

## Discussion

There were 4632 genes differentially expressed in melioidosis and 5045 genes in tuberculosis, thus approximately 20% of the human genome is differentially regulated in each disease. The most prominent pathway in melioidosis was interferon (IFN)-γ and the same was true of tuberculosis. There were no pathways differentially regulated in melioidosis that were not also differentially regulated in tuberculosis, and there was no signature which reliably distinguished melioidosis and tuberculosis.

Berry *et al.* identified an 86-gene signature as being specific for tuberculosis after eliminating differentially regulated genes common to *Streptococcus pyogenes* and *Staphylococcus aureus* infections, and to two auto-inflammatory diseases (systemic lupus erythematosus and Still’s disease). This signature was also present in melioidosis, which is surprising given that all melioidosis patients recruited had acute rather than chronic melioidosis, which is clinically distinct from tuberculosis.

### Interferon-mediated Responses

The IFN-γ pathway was reported as the most prominent pathway identified in gene expression studies of a mouse model of melioidosis [27], and blocking IFN-γ dramatically increases host susceptibility to melioidosis [3]. In human studies, plasma IFN-γ concentrations were high in melioidosis [3], and IFN-γ-mediated responses were also the most prominent feature in a gene expression study of melioidosis in another human cohort [28]. The finding here that this feature is shared with tuberculosis is unsurprising, because IFN-γ responses are crucial for the host response against intracellular pathogens such as *B. pseudomallei* and *M. tuberculosis*. IFN-γ treatment has a role in the management of multidrug-resistant tuberculosis, and adjunctive therapy with IFN-γ is beneficial in a mouse model of melioidosis [29], although its role in clinical melioidosis remains undefined [30].

In their original report on this tuberculosis cohort, Berry *et al.* noted that type 2 IFN-γ responses were prominent, but noted that type 1 IFN-αβ responses were present also [7]. We found that type 1 interferon-αβ responses were just as prominent in melioidosis, but the clinical relevance of this remains to be defined.

Type 1 interferons can be produced by almost any cell type (leukocytes, fibroblasts and endothelial cells) and are induced by a range of bacterial pathogens, whereupon they proceed to modulate the host response in a manner that is as yet incompletely understood [31]. The signalling pathways initiated by type 1 interferons are best described in terms of their activation of signal transducer and activator of transcription (STAT) family members (STAT1 to STAT6) [32], the best studied of which are STAT1 and STAT3. STAT1 activation is dependent on both type 1 and type 2 interferons and results in a pro-inflammatory response, with recruitment of inflammatory cells and the enhancement of antigen presentation [31]. On the other hand, STAT3 activation is a key mediator of IL-10 signalling, and results in inhibition of inflammatory responses and directly inhibits STAT1 activation [31]. The role of STAT4 is less well described, but STAT4 activation may play a role in T helper 1 lymphocyte differentiation, which is an essential part of the host response to intracellular pathogens. Type 1 interferons are also necessary for the production of inducible nitric oxide synthase [33], which is in turn necessary for the clearance of intracellular bacteria. Interestingly, type 1 interferons are able to inhibit IL-1β production and inflammasome assembly by two separate mechanisms: the first is via inhibition of NLRP1 and NLRP3 inflammasomes in a STAT1-dependent manner; the second, is a reduction in pro-IL-1 levels via a STAT3-dependent pathway [34]. It has previously be shown that host response to *B. pseudomallei* is inflammasome-dependent [35].

The role of type 1 interferons in tuberculosis is unclear, since mice deficient in the production of type 1 interferons are better able to control *M. tuberculosis* infections [36], but type 1 interferons also play a non-redundant protective role in the absence of type 2 interferon signaling [37]. The role of type 1 interferons in the pathogenesis of melioidosis remains to be studied.

### PAMP-specific Responses

TLR4 and CD14 are upregulated in both melioidosis and tuberculosis. The classical ligand for TLR4 [38] and for CD14 [39] is lipopolysaccharide (LPS), which would explain this finding for *B. pseudomallei*. TLR4 will recognize heparin-binding haemagglutinin [40], and CD14 will bind lipoarabinomannan [41], both of which are expressed by *M. tuberculosis*.

The pattern recognition receptors TLR5 [25] and NLRC4 [26] both recognize flagellin. No alternative ligand has yet been described for TLR5, so it is more difficult to explain why tuberculosis should apparently induce a flagellin-response. One explanation is that upregulation of pattern recognition receptors is not driven by their ligands. TLR5 expression is induced as part of the type 1 interferon response [42], while NLRC4 is upregulated as part of the TNF-α response [43]. Both pathways are prominent in the host response to melioidosis. In support of this hypothesis, the TLRs are upregulated as a group in both melioidosis (TLR1, TLR2, TLR4, TLR5, TLR6, TLR8 and TLR10) and tuberculosis (TLR2, TLR4, TLR5, TLR6, TLR7, TLR8).

### Limitations and Future Research

Tuberculosis is strongly associated with HIV infection, but melioidosis is not. HIV targets primarily CD4-positive T-lymphocytes and lymphocyte depletion is a feature of all sepsis. Lymphocytes were depleted in both the melioidosis and the TB cohorts, so lymphocyte-related pathways and modules are missing from the whole blood gene expression data of both cohorts, making it difficult to make any comment about the relative role of CD4-positive cells in melioidosis compared to tuberculosis. The whole blood signature was dominated by neutrophils which may also have obscured any lymphocyte signature. Future studies that use purified lymphocytes harvested from melioidosis patients may shed light on this issue.

Microarrays generate large amounts of data that are useful for the development of hypotheses. Our analysis has identified a number of other pathways that are differentially regulated in melioidosis, but which are unstudied to date. Notably, the TRAIL pathway is differentially regulated in melioidosis, but its role remains undefined at present. The glypicans (cell surface proteoglycans) contribute to cell proliferation and growth, both essential processes in the host response to infection. To date, investigations into the role of glypicans have been confined primarily to cancer biology, although glypican-deficient mice are more susceptibility to respiratory infections [44]. In tuberculosis, the glypican network appears to have greater prominence than even the interferon-mediated responses.

### Conclusions

Host responses to melioidosis and TB are dominated by interferon-signalling events, despite the fact that the organisms are unrelated and present completely different cell-surface PAMPs to the host. This is likely because they both stimulate host responses common to intracellular pathogens, and because the expression of pattern recognition receptors is not driven by their ligands, but by cytokine responses (primarily IFN-γ and TNF-α). The 86-gene signature identified by Berry *et al.* clusters melioidosis patients just as effectively as it clusters tuberculosis. It therefore seems likely that whole blood gene signatures will not be able to diagnose tuberculosis in areas where melioidosis and TB are co-endemic, but may find utility when interpreted in combination with clinical features. Further studies using direct comparisons will be required to confirm this finding.
